# Case of large lentigo maligna melanoma of the scalp treated with 5% and 3.75% Imiquimod^☆^^☆☆^

**DOI:** 10.1016/j.abd.2020.08.025

**Published:** 2021-07-12

**Authors:** Miriam Rovesti, Alfredo Zucchi, Claudio Feliciani, Francesca Satolli

**Affiliations:** Department of Dermatology, University of Parma, Parma, Italy

**Keywords:** Dermoscopy, Imiquimod, Melanoma

## Abstract

The paper presents a case of lentigo maligna melanoma of the scalp in an elderly patient treated for the nodular part with surgery and the residual melanoma *in situ* with 5% Imiquimod and subsequently with 3.75% Imiquimod (each concentration for 4 months, 5 times per week), with complete regression of the lesion. 3.75% Imiquimod, which is already used for the treatment of actinic keratosis, could be a useful weapon with the same effectiveness and fewer side effects compared to 5% Imiquimod.

## Introduction

It has been estimated that the 20% of melanomas appear from the head and neck region, among which the 3% appear in the scalp.^1^ melanoma *in Situ* (MIS) is an early variant of melanoma in which the disease is limited to the epidermis. The most common subtype of MIS, lentigo maligna (LM), has a prevalence of 10% to 30% and a 2.2% to 4.7% risk weight of transformation into invasive melanoma or LM melanoma (LMM).^2^

Head and neck melanomas, particularly LM and LMM, have specific dermoscopic features.^1^ Dermoscopy is therefore not only a non-invasive technique that helps distinguishing melanomas from other pigmented and non-pigmented skin lesions but also a valuable tool, which allows an early diagnosis of the tumor and the possibility to follow the lesion over time.

Standard therapy for MIS is surgical excision, but in large lesions, where the anatomic location of the lesion would bring to esthetic and/or functional impairments, complete surgical excision is often unfeasible. Some suggested non-surgical treatments of this residual disease include radiotherapy, cryotherapy and topical Imiquimod.^2^

## Case report

**Figure 1:**
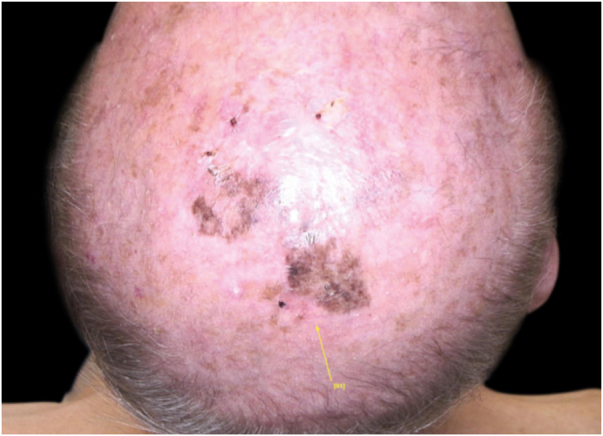
Residual pigmentary lesion after surgery.

A 90-year-old patient underwent surgery for a nodule on the vertex with the outcome of non-ulcered nodular melanoma, which was dermis-infiltrating with 2.9 mm Breslow. Associated to this nodular part, there was an extended flat pigmented lesion, strongly suspected for lentigo maligna (Fig. 1). After a negative TC total body scan and a negative brain MRI, the residual lesion was biopsied and the diagnosis of melanoma *in situ*, lentigo maligna sort, was confirmed.

**Figure 2:**
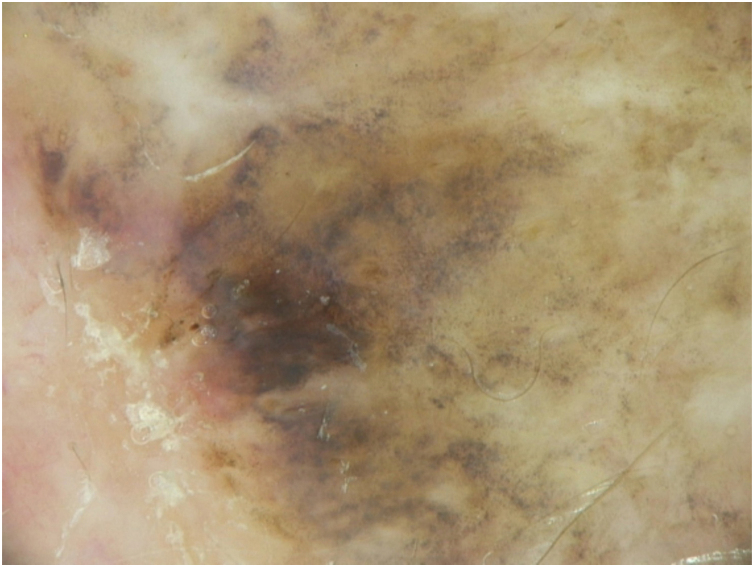
Some dermoscopic features of the lentigo maligna-melanoma *in situ*.

A dermoscopy of the residual lesion shows the dermoscopic criteria of LM: thick pigmented lines around appendageal openings (called rhomboidal structures), asymmetric pigmentation with angulate brown, grey lines and grey dots, dark blotches, and obliterated hair follicles (some details of the dermoscopic aspect are shown in Fig. 2).

**Figure 3:**
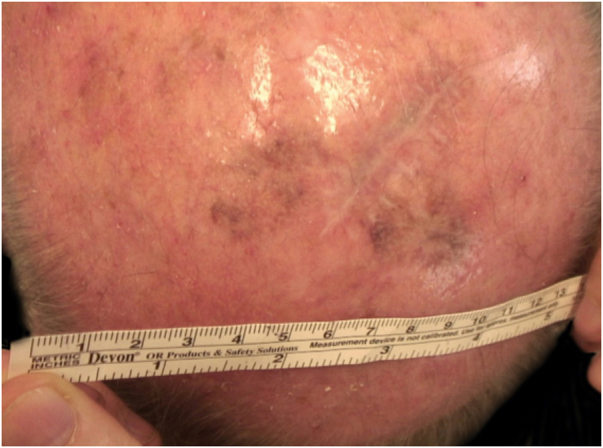
50% resolution of the lentigo maligna after 4 months of 5% Imiquimod.

**Figure 4:**
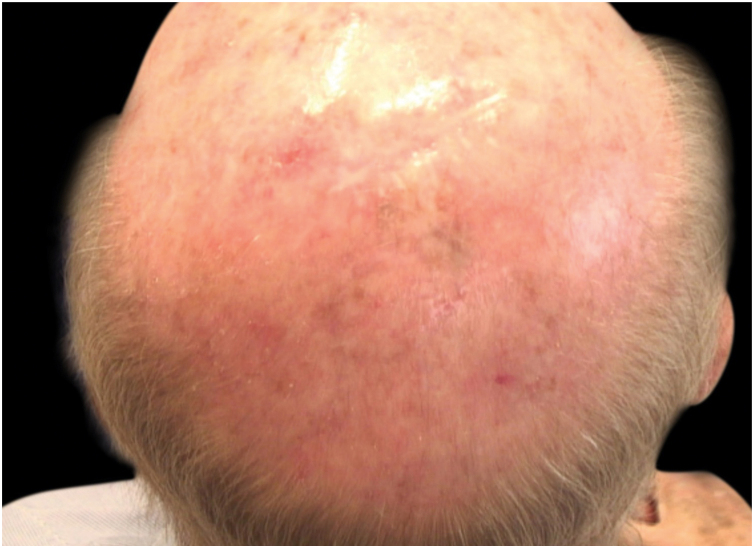
Almost complete resolution of the lentigo maligna after 3 months of 3.75% Imiquimod.

Considering the lesion extent, the histological variant (lentigo maligna sort), and the patient’s age, it has been decided to treat it with 5% Imiquimod 5 times per week for 4 months (with 50% resolution of the melanoma *in situ*: Fig. 3), and subsequently with 3.75% Imiquimod for other 4 months, without occlusion and without interruption between the two sessions, with complete resolution of the MIS (Fig. 4, taken after 3 out of 4 months of 3.75% Imiquimod therapy).

## Discussion

Lentigo maligna is an *in situ* variant of melanoma that appears as a slowly enlarging brown to grey-black pigmented and sometimes amelanotic macule on chronically sun-exposed skin. In particular, in patients who are older than 45 years old, the incidence of LM and LMM is increasing.^3^

For the diagnosis and management of LM and LMM, dermoscopy is an indispensable tool as suggested by literature; as a matter of fact, dermoscopy not only aids the diagnosis – helping to find the most appropriate area to biopsy, to evaluate the margins of the tumor, etc. – but allows a non-invasive follow up during the treatments, particularly the topical ones, by confirming the therapeutic efficacy – in other words, by confirming that the tumor criteria are not to be found anymore.^4^ In the studied patient, a dermoscopy evaluation every two months allowed us to monitor and continue the topical therapy.

Speaking of the treatments, the international guidelines’ recommendations based on expert opinions state that surgical excision with at least a 5 mm margin is the first-choice therapy.^5^ Nonetheless, the surgical management of LM can be challenging in cases of large lesions, for various reasons: reconstructive procedures may be needed after excision and most of the patients with LM are elderly, thus they may be frail and suffer from comorbidity – as in the studied patient.

Over the past 15 years, imiquimod cream gained attention as an off-label, topical and non-invasive treatment modality for LM. Topical imiquimod is a synthetic imidazoquinoline amine with toll-like receptor 7 agonist properties, and it is able to increase the production of inflammatory cytokine and chemokines. Imiquimod also exerts its effect on tumor cell apoptosis through activation of the caspase pathway; moreover, it has angiogenic effects through the downregulation of fibroblast growth factor and the upregulation of inhibitors of angiogenesis.^2^ According to the literature, 5% Imiquimod is effective in the treatment of external condylomata acuminata, cutaneous warts, superficial basal cell carcinoma, and actinic keratosis.^2^ However, recent studies have demonstrated similar efficacy and have reported lower complications using 3.75% Imiquimod for the treatment of actinic keratosis.^6^, ^7^

Regarding melanoma *in situ*, the authors would like to underline that imiquimod is currently not approved in the treatment of LM and LMM, however, more and more papers support its effectiveness for the treatment of lentigo maligna, as a single treatment or after surgery and cryotherapy.^8^ In literature are described cases of no recurrence of LM after treatment with Imiquimod 5% and also papers describing recurrences after this kind of treatment, this is also due to a complete clinical resolution did not always correlate with histological clearance and clinical suspicion for the residual disease is not always confirmed histopathologically.^9^, ^10^

Unfortunately, a standard treatment schedule does not exist. A systematic review on the role of imiquimod in LM and LMM concludes that 6–7 applications of 5% Imiquimod per week, with at least 60 applications, show the highest probability of a complete clinical and histological clearance of LM.^3^

In the studied patient, the authors preferred the application 5 times a week of the 5% composition, for approximately 80 applications, and sequentially other 80 applications of the 3.75% Imiquimod (5 times per week), in order to reduce the side effects of the treatment (local inflammation) and to evaluate the equal effectiveness of a reduced concentration of the drug. In the studied patient, both the 5 and the 3.75 Imiquimod percentage demonstrated its effectiveness and led to a complete resolution of the LM in approximately 160 sessions (80 applications with 5% Imiquimod and 80 sessions with 3.75% Imiquimod).

In conclusion, in the authors’ experience, the topical 5% and the 3.75% Imiquimod are two valid options for lentigo maligna in patients unfit or not willing to undergo surgery or radiotherapy. The 3.75% Imiquimod can be a valid choice used sequentially after 5% Imiquimod or as the first treatment option, in order to reduce the side effects of the drug. To date both the concentrations of Imiquimod are unfortunately off-label treatments, thus they require informed consent by the patient for their use.

In the future, more and more patients with LM should receive by dermatologists a personalized treatment plan and, in case of inoperable lesions, a specific treatment schedule of Imiquimod, considering the initial clinical presentation, the host factors influencing immune response, the side effects, and the histological results (pre and post-treatment).

## Financial support

None declared.

## Authors’ contributions

Miriam Rovesti: Effective participation in the manuscript; critical literature review; preparation and writing of the manuscript.

Alfredo Zucchi: Intellectual participation in the therapeutic management of the studied case; manuscript critical review.

Claudio Feliciani: Intellectual participation in the therapeutic management and final approval of the latest version of the paper.

Francesca Satolli: Effective participation in the manuscript; intellectual participation in the therapeutic management of the case; manuscript critical review; final approval of the latest version of the paper.

## Conflicts of interest

None declared.
